# Mapping of immunogenic and protein-interacting regions at the surface of the seven-bladed β-propeller domain of the HIV-1 cellular interactor EED

**DOI:** 10.1186/1743-422X-5-32

**Published:** 2008-02-27

**Authors:** Dina Rakotobe, Sébastien Violot, Saw See Hong, Patrice Gouet, Pierre Boulanger

**Affiliations:** 1 Laboratoire de Virologie & Pathologie Humaine, Université Lyon I & CNRS FRE-3011, Faculté de Médecine Laennec, 7 rue Guillaume Paradin, 69372 Lyon Cedex 08, France; 2 Laboratoire de BioCristallographie, IBCP, Instititut Fédératif de Recherche IFR128 BioSciences Lyon-Gerland, 7 passage du Vercors, 69367 Lyon Cedex 07, France; 3 Laboratoire de Virologie Médicale, Centre de Biologie & Pathologie du Pôle Est, Hospices Civils de Lyon, 59 Boulevard Pinel, 69677 Bron Cedex, France

## Abstract

**Background:**

The human EED protein, a member of the superfamily of *Polycomb *group proteins, is involved in multiple cellular protein complexes. Its C-terminal domain, which is common to the four EED isoforms, contains seven repeats of a canonical WD-40 motif. EED is an interactor of three HIV-1 proteins, matrix (MA), integrase (IN) and Nef. An antiviral activity has been found to be associated with isoforms EED3 and EED4 at the late stage of HIV-1 replication, due to a negative effect on virus assembly and genomic RNA packaging. The aim of the present study was to determine the regions of the EED C-terminal core domain which were accessible and available to protein interactions, using three-dimensional (3D) protein homology modelling with a WD-40 protein of known structure, and epitope mapping of anti-EED antibodies.

**Results:**

Our data suggested that the C-terminal domain of EED was folded as a seven-bladed β-propeller protein. During the completion of our work, crystallographic data of EED became available from co-crystals of the EED C-terminal core with the N-terminal domain of its cellular partner EZH2. Our 3D-model was in good congruence with the refined structural model determined from crystallographic data, except for a unique α-helix in the fourth β-blade. More importantly, the position of flexible loops and accessible β-strands on the β-propeller was consistent with our mapping of immunogenic epitopes and sites of interaction with HIV-1 MA and IN. Certain immunoreactive regions were found to overlap with the EZH2, MA and IN binding sites, confirming their accessibility and reactivity at the surface of EED. Crystal structure of EED showed that the two discrete regions of interaction with MA and IN did not overlap with each other, nor with the EZH2 binding pocket, but were contiguous, and formed a continuous binding groove running along the lateral face of the β-propeller.

**Conclusion:**

Identification of antibody-, MA-, IN- and EZH2-binding sites at the surface of the EED isoform 3 provided a global picture of the immunogenic and protein-protein interacting regions in the EED C-terminal domain, organized as a seven-bladed β-propeller protein. Mapping of the HIV-1 MA and IN binding sites on the 3D-model of EED core predicted that EED-bound MA and IN ligands would be in close vicinity at the surface of the β-propeller, and that the occurrence of a ternary complex MA-EED-IN would be possible.

## Background

Human EED protein, the human ortholog of the mouse embryonic ectoderm development (*eed*) gene product, is a member of the superfamily of WD-40 repeat proteins which belongs to the highly conserved *Polycomb *group (*Pc*G) family of proteins [1-7]. The human EED protein has been found to interact with several cellular proteins in both cytoplasmic and nuclear compartments. At the inner side of the plasma membrane, EED interacts with the cytoplasmic tail of integrin β7 subunit [8], a domain involved in major integrin functions [9,10]. Within the nucleus, EED participates in Polycomb Repressive Complexes (PRCs), multiprotein edifices which have been identified in *Drosophila *and in mammals (reviewed in [11]). Several types of PRCs have been described and referred to as PRC1, PRC2 and PRC3 [12]. PRC2/3 content includes, among other components, EED, EZH2, SUZ12 and RbAp46/48 [12-14].

In the context of HIV-1-infected cells, EED has been found to interact with three viral proteins, the structural protein matrix (MA) [15], the enzyme integrase (IN) [16] and the regulatory protein Nef [17]. These interactions involved the C-terminal domain of EED, or EED core, common to the four isoforms. It has been suggested that the nuclear depletion of EED which resulted from the EED-Nef interaction occurring at the plasma membrane of HIV-1-infected cells would be responsible for the release of an EED-mediated transcriptional block and for an indirect transcriptional activation of the virus [17]. This hypothesis was consistent with the reported functions of *Pc*G proteins, which act as transcriptional repressors of homeotic genes (reviewed in [11,18-20]), and contribute to the maintenance of the silent state of chromatin in upper eukaryotes [21]. It was also consistent with the finding that HIV-1 preferentially integrates into transcriptionally active regions of the host genome [22-25]. Thus, at the early phase of the HIV-1 life cycle, EED might play a role in targeting the regions of proviral DNA integration into the host chromatin. At the late steps of the virus replication cycle, we found that overexpression of isoforms EED3 and EED4 had a significant negative effect on virus production, and that virus assembly and genome packaging were the major targets of this EED inhibitory activity [26].

The finding that EED was an interactor of three HIV-1 components and an intracellular factor possibly involved in antiviral innate immunity prompted us to analyse the three-dimensional (3D) structure of EED. Crystallogenesis of EED was therefore undertaken to better understand the nature of the multiple interactions and functions of EED in the HIV-1 life cycle. Unfortunately, none of our attempts to obtain diffracting crystals of EED alone, or in complex with its viral partners MA, IN or Nef was successful, and we therefore analyzed the 3D structure of EED using indirect approaches. They consisted of (i) three-dimensional modelling based on computer-assisted methods of sequence alignment and determination of homology with a prototype of seven-bladed β-propeller protein previously crystallized [27,28]; (ii) mapping of accessible regions of the EED protein, using anti-EED antibodies and a phage display technique.

During the completion of this work, crystallographic data of the EED protein core, co-crystallised with a peptide from the N-terminal domain of EZH2, was deposited in the protein data bank (PDB code #2QXV) [29] and later published [30]. Our predictive model determined by indirect methods was in good consistency with the crystal structure of EED, except for the region 267–295 which comprises a unique α-helix facing a short β-strand in the crystallographic structure. Major immunogenic regions in EED were found to correspond to flexible loops and β-strands which were accessible at the surface of the β-propeller. In addition, EED modelling suggested that HIV-1 MA and IN bound to two contiguous sites forming a continuous protein-interacting domain localized in a groove running along the lateral face of the EED β-propeller.

## Results and Discussion

### EED Crystallogenesis

**Figure 1:**
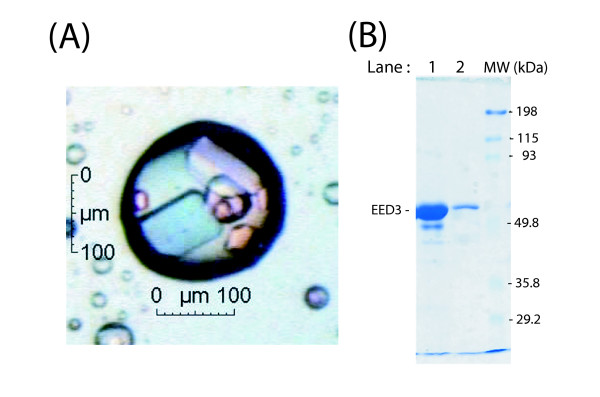
**Crystallogenesis of histidine-tagged isoform 3 of EED**. **(A)**, Platelet-like crystals of EED3-H_6 _protein of 441 residues, obtained in suspended drop in 0.1 M MES buffer pH 6.0, 40 % MPD. **(B)**, Solubilization of the crystals and analysis by SDS-PAGE and Coomassie blue staining. Lane 1 : solution of purified EED3-H_6 _(10 mg/mL) used for crystallogenesis (protein load : 50 μg). Lane 2 : protein content of solubilized single crystal. MW : markers of protein molecular mass, indicated in kilodaltons (kDa) on the right side of the panel.

The coding sequence for the His-tagged EED protein of 441 residues representing isoform 3 (EED3-H_6_) was expressed in *E. coli *[16]. EED3 corresponded to the sequence spanning residues Met95-Arg535 in the EED1 isoform [12]. EED3-H_6 _protein was found to be highly soluble and was purified to homogeneity, using affinity chromatography followed by a gel filtration step. Solutions of EED3-H_6 _titrating 5 to 10 mg/ml were subjected to more than a thousand of different conditions for crystallization. EED3-H_6 _protein crystals, appearing as thin platelets of 0.1 × 0.07 × 0.01 mm^3^, were observed after 30 days at 19°C under certain buffer conditions (0.1 M MES buffer, pH 6.0, 40 % MPD ; Fig. 1A). One single crystal was removed from the well-buffer, washed and dissolved in SDS-sample buffer. SDS-PAGE analysis showed that this crystal was really constituted of EED3-H_6 _protein (Fig. 1B; lane 2). However, the crystals obtained under these conditions failed to generate X-ray diffraction patterns. We then tried to co-crystallise EED3-H_6 _with its viral protein partners MA, IN or Nef, respectively, but all these attempts were unsuccessful. We then used alternative methods for structure determination of EED, as described below.

### 3D-modelling of the EED core domain

**Figure 2:**
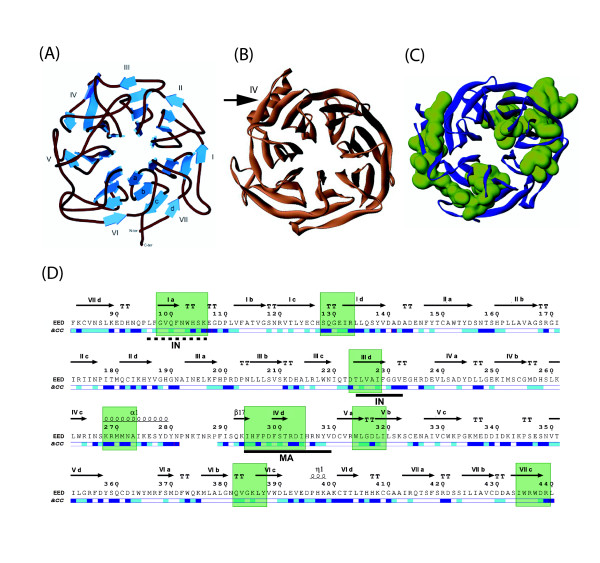
**Structural models and immunogenic regions of EED isoform 3**. **(A)**, Seven-bladed β-propeller model of the EED core domain, based on sequence homology with the beta subunit of the bovine G protein (Gβ ; [27, 28]). Shown is a ribbon representation of the polypeptide backbone atoms of EED3 isoform (amino acid residues 84–441), with secondary and tertiary structures of the different β-blades. **(B)**, 3D-model of the EED3 seven-bladed β-propeller, deduced from crystallographic data (modified, from [30]). The black arrow indicates the major difference between our putative model (A) and the crystal model (B), consisting of the α1 helical region facing the β-strand β17 in β-blade IV. **(C)**, Position of immunogenic epitopes (depicted in green) on the 3D-model of EED polypeptide backbone (represented in blue). **(D)**, Primary and secondary structures of EED3, deduced from crystallographic data [30]. The amino acid sequence was numbered according to the accepted nomenclature [12] : Met95 in EED1 isoform represented Met1 in EED3 ; thus, the C-terminal residue L440 in EED3 corresponded to L535 in EED1. Regions in β-strand structure are represented by horizontal arrows, with reference to the blade number and β-strand letter a, b, c or d ; α-helices are represented by spirals, and turns by TT. Helical regions marked α1 and η1, and the β-strand region marked β17, were structurized domains of EED which were unique among representatives of WD-40 proteins. The relative accessibility of each residue (*acc*) in the 3D structure was extracted from the dictionary of protein structure [45], and indicated as coloured bars under the sequence with the following colour code : dark blue, highly accessible ; light blue, accessible ; white, buried. Discrete regions recognized by anti-EED IgG are indicated by green boxes. The binding sites of HIV-1 matrix protein (MA) and integrase (IN) are underlined by solid black lines.

Seven repeats of a canonical WD-motif have been identified in the C-terminal core of the EED protein, shared by the four isoforms EED1, EED2, EED3 and EED4 [12,15]. It was therefore possible to build a three-dimensional model for the C-terminal core domain of EED spanning residues 84–441 (roughly corresponding to the EED3 isoform), using homology modelling by sequence alignment and homology with protein(s) of known structures, and assessment of accessible motifs and epitopes at the surface of the EED protein. The template used was the β subunit of the bovine signal-transducing G protein (Gβ), of which crystal structure has been determined [27,28]. However, due to the limited degree of identity between their primary structures (only 20 % amino acid residues identical between EED3 and Gβ), the sequence alignment of both proteins was manually optimized to improve the correspondence between consensus residues in the WD repeats. The model obtained for EED3 corresponded to a typical seven-bladed β-propeller structure (Fig. 2A). Each WD-40 repeat was folded as 3 β-strands referred to as a, b and c, respectively. The sequence connecting every WD-40 repeat also folded as an additional β-strand, called d. Thus, a WD-40 repeat formed a structural unit made of 4 antiparallel β-strands referred to as β-blade, and the seven β-blades defined in EED were folded as a β-propeller structure (Fig. 2A).

Our β-propeller model was confirmed by the refined crystal model of the EED core domain recently published [30], and depicted in Fig. 2 (panels B and D). EED and EZH2 proteins are partners involved in PRC2/3 complexes, along with SUZ12 and RbAp46/RbAp48 [13]. The proposed structure represented the co-crystallized complex of a fragment of the N-terminal domain of EZH2 (residues 39 – 68) with the C-terminal domain of EED (residues 82 – 440). EED-EZH2 interaction took place via the insertion of both ends of the EZH2 α-helical peptide into two peptide-binding hydrophobic pockets in EED formed by the side chains of V112, L123, W152 and P161, and by residues L318, L353, L391 and P396, respectively [30]. The 3D structure reconstructed from crystallographic data was globally similar to our 3D-model of seven β-bladed propeller, with three exceptions. In β-blade IV, there were two structures at the junction of β-strands IVc and IVd that were unique among representatives of WD-40 proteins, (i) an α-helix encompassing region 267–280 (α1) and (ii) an outer β-strand referred to as β17. (iii) In β-blade VI, a short 3_10_-helix (termed η1) was found on the N-terminal side of β-strand VId (Fig. 2D).

### Surface-exposed regions in the seven-bladed β-propeller domain of EED

#### Theoretical considerations

An important feature of the β-propeller structure of the EED core was that most of the accessible surfaces should be confined to the outer β-strands d, and to flexible loops connecting β-strands of the same blade (Fig. 2D). These accessible regions would be potential sites of protein-protein interaction, as shown by X-ray diffraction analysis of protein complexes involving other β-propeller proteins [31,32]. E.g. in the case of bovine Gβ protein, several residues belonging to loops d-a and b-c were found to be involved in hydrophobic contacts with the α subunit [32]. These accessible regions would also contain putative immunogenic epitopes, responsible for the induction of EED antibodies in animals in response to administration of human EED. The next experiments were designed to test this hypothesis.

#### Mapping of accesible regions in EED using anti-EED antibodies and phage biopanning

We raised in rabbit a highly reactive and specific antiserum against affinity chromatography-purified, His-tagged EED3 isoform also used for crystallization trials. IgG were isolated from this antiserum and used in a screen with recombinant phages [15,16]. We reasoned that anti-EED IgG immobilized on a solid support would preferentially bind to phages which are mimotopes of accessible and antigenic regions of EED [33,34]. The phages selected on these anti-EED antibodies mapped to eight discrete regions on EED, numbered 1 to 8 (Fig. 2C, D). These regions spanned residues 98–106 (1), 128–133 (2), 223–228 (3), 268–273 (4), 294–305 (5), 314–319 (6), 382–387 (7) and 434–439 (8), respectively. Five of these regions, n° 2, 4, 5, 6 and 7, corresponded to predicted accessible loops separating β-strands in the EED 3D-structure (Fig. 2C, D). Interestingly, the N-terminal portion of the α-helix K268-D279 coincided with the immunogenic region 4 that we identified (Fig. 2D).

The question raised however, for the accessibility of three regions, numbered 1, 3 and 8, which were partially or totally folded as β-sheet (Fig. 2B–D). Region 1 overlapped with β-strand Ia and the adjacent loop a-b forming the junction with β-strand Ib (Fig. 2B–D). Its motif 103-WHS-105 was included in the EZH2 binding site, and was accessible in a groove oriented towards the lower face of the propeller [30]. Likewise, the reactivity of region 3, which coincided with β-strand IIId, was in good consistency with the 3D-model, as it was oriented outwards and accessible at the surface of the β-propeller. However, our data concerning region 8 were more intriguing : this region corresponded to the β-strand VIIc which was close to the C-terminus and was accessible to antibodies in our experimental screening. This suggested that EED in solution adopted a 3D structure which was less tightly closed than shown in the 3D model. Thus, our mapping of major immunogenic regions of EED was in good consistency with the position of accessible loops and surface exposed portions of β-stands predicted by the EED 3D-model.

#### 3D structure and protein interacting regions in the EED core domain

The binding site of the HIV-1 MA protein has been mapped to position 294–309 on the linear sequence of EED [15]. The newly established conformation of this region implied that the region of interaction with the MA protein was not only confined to the flexible loop IVd-Va on the upper face of the β-propeller, but also included the short, rigid β-strand IVd and the neighboring loop IVd-Va, located on the lateral face of the β-propeller. This was not contradictory to our mapping of the MA binding site on the EED linear sequence, since β-strands d were the most exposed β-strands at the periphery of the β-propeller (Fig. 2D). Of note, the upper face of the β-propeller was narrower in surface, compared to its lower face.

However, there was some ambiguity in the determination of the IN binding domain in EED, as two potential binding sites (bs) were identified by phage display, one at position 96–105 (bs1), the other one at position 224–232 (bs2) [16]. In the light of the EED crystal structure, it appeared that in bs1, residues 97–102 were buried in the β-propeller central tunnel, and amino acids 103–105 were part of the groove on the lower face of the EED β-propeller which homed the N-terminal fragment of EZH2 in co-crystals [30]. F96 was the only residue of bs1 which was oriented upwards and accessible on the top of EED. By contrast with bs1, bs2 mapped to the β-strand IIId and the neighbouring turn included in loop IIId-IVa (Fig. 2D). This region lied at the periphery of the β-propeller and was therefore highly accessible, as determined from crystallographic data (Fig. 2B, D).

It was therefore difficult to conceive how one single IN molecule could bind simultaneously to bs1 and bs2, as these sites were far from each other and in different orientation with respect to the β-propeller plane. Although the possibility existed that one molecule of EED would bind to two IN molecules (e.g. dimeric or tetrameric forms of IN), this was unlikely for the following reasons : (i) only one single EED-binding site has been identified in the HIV-1 IN sequence [16], and it is unlikely that the same IN motif would bind to two different sequences in EED ; (ii) mutant EED-103A3, in which the tripeptide motif 103-WHS-105 was replaced by the tripeptide AAA, was still binding to IN with significant efficiency [16]. Taken together, these results suggested that region 224–232 (bs2) was the most probable and unique binding site for IN on EED.

**Figure 3:**
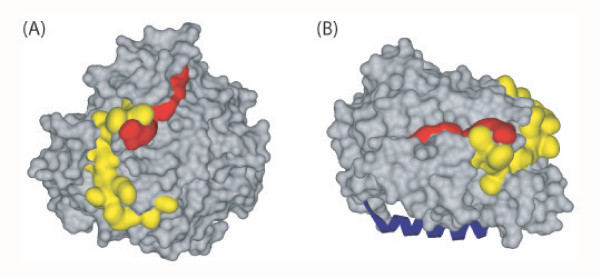
**Surface representation of the β-propeller domain of EED and protein-interacting regions**. The binding residues of HIV-1 proteins are represented with the following colour code : yellow for the matrix protein (MA), red for integrase (IN). **(A)**, Top view of the β-propeller showing the MA and IN binding sites laterally oriented. Note the absence of overlapping between the MA and IN binding sites, which form a continuous binding groove. **(B)**, Side view of the β-propeller showing the MA+IN-binding groove on the lateral face, and the position of the EZH2 α-helical peptide 39–68 (represented in blue), bound to the EZH2-binding pocket facing downwards.

Although the IN and MA binding sites were found to be located at significant distance from each other on the EED linear sequence (224–232 and 294–309, respectively; Fig. 2D), they appeared to be in close vicinity in the 3D structure : both were located on the lateral face of the EED β-propeller, as shown by surface representation, but they did not overlap (Fig. 3A). This was corroborated by the absence of competition between MA and IN for binding to EED3-H_6 _protein in vitro in histidine pull-down assays (data not shown). In addition, the possibility of occurrence of ternary complex involving EED, MA and IN has previously been suggested by their colocalization observed by immuno-electron microscopy of HIV-1-infected cells at early steps of the virus life cycle [16].

The EZH2 binding groove, which was oriented downwards with respect to the EED β-propeller plane, was totally independent of the continuous MA-IN binding groove (Fig. 3B). Interestingly, although α-helices represent privileged domains of protein-protein interaction, none of the newly identified helices in the EED core, α1 or η1, represented binding domains of known cellular or viral partners of EED, e.g. EZH2 [30], MA [15], or IN [16].

## Conclusion

The refined structural model of the EED C-terminal core as a seven-bladed β-propeller determined from crystallographic data provided structural support to our mapping of immunogenic epitopes recognized by our anti-EED polyclonal antibodies, and of the binding sites of HIV-1 MA and IN [15,16]. Several immunoreactive regions coincided with the MA, IN and EZH2 binding sites, confirming the accessibility of these regions at the surface of EED. According to the EED 3D-model, the domain of interaction with the HIV-1 MA protein would be localised on the lateral face of the β-propeller, and be comprised of two loops separated by the short β-strand IVd (Fig. 2 and Fig. 3). The region of interaction with IN would be assigned to β-strand IIId and its neighboring turn, also located on the peripheral area of the β-propeller. When represented on the surface of the EED molecule, the two discrete prints of MA and IN interaction were contiguous but did not overlap, and formed a continuous protein-interacting groove running along the lateral face of the EED β-propeller. This groove slightly opened towards the lower face of the β-propeller (Fig. 3). The absence of overlapping of the MA and IN binding sites and the possible occurrence of ternary complex involving EED, MA and IN raised the issue of the biological parameters of a simultaneous binding of EED to MA and IN, and of the role that such a ternary complex might play in the HIV-1 life cycle.

## Methods

### Plasmids, proteins and cells

Plasmids coding for GST-fused or His-tagged proteins EED, MA, IN and Nef and protein expression in bacterial cells have been described in previous studies [15,16,26,35].

### EED crystallogenesis

The commercial kits used (Crystal screen 1 and 2 ; Grid Screen Ammonium sulfate ; Grid Screen Sodium Chloride ; Grid Screen MPD ; Grid Screen PEG 6000 ; Grid Screen PEG/LiCl ; Natrix ; SaltRX ; Index & PEG; Ion Screen) were purchased from Hampton Research (Aliso Viejo, CA, USA) and NeXtal (PEG Suite ; Anions & Cations ; Qiagen SA). The screen was carried out in 96-well plates (Greiner Bio-One GmBH, Divison BioScience, Les Ulis, Courtaboeuf, France), using the hanging-drop vapor-diffusion method. The drops were generated using the Mosquito^® ^Crystal technology (TTP LabTech Ltd, Melbourne, Hertfordshire, UK). Crystals were obtained by the vapor diffusion method from a solution containing 0.1 M 2-(N-morpholino)ethanesulfonic acid (MES) buffer, pH 6.0, 40 % 2-Methyl-2,4-pentanediol (MPD) at a temperature of 292 K. A 1:1 ratio of protein to reservoir solution was used.

### Protein purification

Isolation of His-tagged proteins from bacterial cell lysates was carried out as follows. *E. coli *BL21 DE3 transformed with pT7.7 plasmid [16] were lysed by resuspension in TBS containing lysozyme (1 mg/mL) and a cocktail of protease inhibitors (Roche Diagnostics Corp., Meylan, France), with five cycles of ultrasonication (20 sec each). Cell debris were removed by centrifugation at 10 krpm for 30 min at 4°C in the Biofuge centrifuge (Heraus, Kendro Laboratory Products, IMLAB Sarl, Lille, France). Affinity purification of His-tagged protein was performed on HiTrap column (1 mL total volume ; GE Healthcare Bio-Siences, Saclay, France). The column was first loaded with Ni_2_^+ ^(0.1 M Ni_2_SO_4_) prior to affinity chromatography, using a high-performance liquid chromatography (HPLC) system (BioLogic DuoFlow ; Bio-Rad France, Marnes-la-Coquette, France). Proteins were adsorbed in 50 mM Tris-HCl buffer, pH 7.5, 150 mM NaCl (TBS) containing 20 mM imidazole, and eluted with TBS containing 1 M imidazole. Further purification was achieved by gel filtration chromatography, using a Superdex-200 column (GE Healthcare Bio-Sciences, Saclay, France) equilibrated in TBS buffer.

#### Antibodies and immunological analysis

Anti-EED rabbit antiserum was laboratory-made. Affinity chromatography-purified, His-tagged EED3 isoform was used as the immunogen. Anti-oligohistidine tag polyclonal antibody was purchased from Qiagen SA (Courtaboeuf, France). For isolation of anti-EED IgG, rabbit antiserum against EED (1 mL) was precipitated by ammonium sulfate at 33 % saturation, and pH 6.5. The IgG precipitate (12–15 mg) was resuspended in TBS (1 mL) and adsorbed on protein G-Sepharose gel. IgG elution was carried out with two gel volumes of 0.1 M Tris-glycine pH 2.2, and the eluate dialyzed against TBS. Proteins were analyzed by electrophoresis in SDS-containing 12 % polyacrylamide gels in the discontinuous Laemmli's buffer system (SDS-PAGE) and Coomassie blue staining, or Western blotting using the above-mentioned antibodies, as previously described [16,26].

### Phage biopanning

Biopanning of the 6-mer phage library and the ligand elution technique have been described in detail in previous studies [15,16,33,34,36]. In brief, for identification of antigenic regions on the EED protein, recombinant bacteriophages were adsorbed onto anti-EED IgG coated on plates. After extensive rinsing, phages were recovered by three successive cycles of acid buffer elution, followed by final elution by affinity chromatography-purified EED-His_6 _protein used as competing ligand [33]. Phagotopes were determined by DNA sequencing.

### Protein homology modelling

The choice of the protein print for EED was determined by sequence comparison using the CLUSTALW program [37] and the PDB [29]). The beta chain of the bovine G protein (Gβ), a WD motif-containing protein, was then obtained (PDB code #1TBG). After preliminary sequence alignment of EED with Gβ, alignment was optimized using the following programs : MLRC [38], DSC [39] and PHD [40], all of them available on the NPS@ server [41]. The construction of the 3D-model of EED from the Gβ structure was carried out by substitution of the amino acid side-chains using the CALPHA program [42]. Reorientation of the side-chains as well as construction of reinserted polypeptide chain fragments were both performed using the TURBO-FRODO program [43]. Final optimization of the EED 3D-model was achieved using the conjugated gradient method and the CNS program [44].

## Competing interests

The author(s) declare that they have no competing interests.

## Authors' contributions

SV and DR performed the laboratory work and contributed equally to this study. PB and SSH conceived the strategies and designed the experiments. PG contributed to EED homology modelling and data analysis. PB wrote the manuscript. All authors read and approved the final manuscript.
